# Prognostic value of interleukin-33, sST2, myeloperoxidase, and matrix metalloproteinase-9 in acute aortic dissection

**DOI:** 10.3389/fcvm.2022.1084321

**Published:** 2023-01-06

**Authors:** Yu Jia, Dongze Li, Jing Yu, Wenli Jiang, Yi Liu, Fanghui Li, Wentao Li, Rui Zeng, Xiaoyang Liao, Zhi Wan

**Affiliations:** ^1^General Practice Ward/International Medical Center Ward, General Practice Medical Center, West China Hospital, Sichuan University, Chengdu, China; ^2^Department of Emergency Medicine and National Clinical Research Center for Geriatrics, Disaster Medicine Center, West China School of Medicine/West China Hospital, Sichuan University, Chengdu, China; ^3^Institute of Biomedical Engineering, West China School of Basic Medical Sciences and Forensic Medicine, Sichuan University, Chengdu, China; ^4^Department of Cardiology, West China Hospital, Sichuan University, Chengdu, China

**Keywords:** acute aortic dissection, interleukin-33, ST2, myeloperoxidase, matrix metalloproteinase-9, mortality

## Abstract

**Background and purpose:**

Acute aortic dissection (AAD) is a life-threatening cardiovascular emergency. Both neutrophil granzyme and interleukin (IL)-33/ST2 systems have proven to be effective diagnostic markers for AAD. This study aimed to investigate the relationship between plasma IL-33, soluble suppression of tumorigenesis-2 (sST2), myeloperoxidase (MPO), and matrix metalloproteinase (MMP)-9 levels at admission and all-cause mortality in patients with AAD.

**Methods:**

A total of 155 patients with AAD were enrolled from the Prospective Evaluation of Acute Chest Pain (PEACP) study. Plasma concentrations of IL-33, sST2, and MMP-9 were measured using an enzyme-linked immunosorbent assay, and MPO was detected using a chemiluminescence immunoassay. Aortic anatomical parameters were measured using CT radiography. The primary endpoint was all-cause mortality rate.

**Results:**

The median age of the patients was 55 years, and 96 (61.9%) were diagnosed with type A-AAD. After adjusting for confounding factors, the highest tertiles of IL-33, sST2, MPO, and MMP-9 had hazard risks of 0.870 (95% CI: 0.412–1.836, *P* = 0.714), 3.769 (95% CI: 1.504–9.446, *P* = 0.005), 4.689 (95% CI: 1.985–11.076, *P* < 0.001), and 4.748 (95% CI: 1.763–12.784, *P* = 0.002), respectively, compared to the lowest tertile. Pearson’s correlation analysis revealed a significant correlation between these markers (*P* < 0.001). Moreover, sST2, MPO, and MMP-9 levels had a significant positive correlation with aortic diameter and pseudolumen area (*P* < 0.001).

**Conclusion:**

The biomarkers sST2, MPO, and MMP-9 were independently associated with mortality in patients with AAD. The significant correlation between these biomarkers suggests a pathogenic role for the IL-33/ST2/neutrophil granzyme system in patients with AAD.

## 1. Introduction

Acute aortic dissection (AAD) is a life-threatening cardiovascular emergency with high morbidity and mortality rates. The type A dissection involves the ascending aorta, and type B is limited to the descending aorta according to the Stanford classification. The in-hospital mortality rate of patients with AAD is as high as 27.4% (1). The risk of death in patients with AAD increases by 1–3% for every hour of delayed diagnosis and treatment (2), and timely treatment can reduce the overall mortality to 13–22% (3–5). Therefore, identifying the risk factors affecting the prognosis of AAD as well as high-risk AAD patients has been the focus of emergency medical staff in recent years.

At present, the early evaluation of AAD mainly depends on imaging anatomical parameters (including diameter of aorta, area of false cavity, etc.) (6) and mechanical indices (such as the hemodynamic and stress state of the vascular wall) (7, 8). However, computed tomography (CT) and magnetic resonance angiography are not routine examination procedures, and most hospitals lack these examination equipment. Blood biomarkers are ideal, minimally invasive bedside monitoring indices. Exploring biomarkers related to the anatomical parameters of the dissecting aorta will contribute to the early risk stratification and prognosis evaluation of AAD and even provide new insights for intervention targets.

Neutrophils are the most abundant circulating leukocytes in the human body. It has been proven that as soon as AAD occurs, neutrophils proliferate and infiltrate, mediating collagen degradation, and adventitia rupture (9). Our previous studies have also shown that elevated neutrophil counts are associated with a higher risk of death in patients with AAD (10). The pathophysiological mechanism of neutrophils mainly depends on their granular granzymes such as myeloperoxidase (MPO) and matrix metalloproteinases (MMPs) (11). Granular granzymes are widely expressed in circulation and have a confirmed association with cardiovascular and aortic diseases (12, 13). Importantly, the cytokines that induce the release of these granular granzymes may also be related to AAD.

Interleukin (IL)-33 is a recently discovered cytokine in the IL-1 family that has been proven to be associated with aortic lesions. Furthermore, circulating soluble suppression of tumorigenesis-2 (sST2), a decoy receptor of IL-33, was shown to be significantly elevated in the early stages of AAD and thus may accurately diagnose AAD in patients with chest pain (14). Moreover, an increasing number of studies have reported the regulatory effects of IL-33 on neutrophils. These include activation, amplification of oxidative stress, and inflammatory response (15–17), and promotion of the formation of neutrophil extracellular traps (18–20).

Thus, we speculated that circulating neutrophil-related inflammatory markers (IL-33, sST2, MPO, and MMP-9) may be related to the severity of AAD and predict the risk of death. To date, few studies have reported the relationship between these inflammatory markers and anatomical parameters of the dissected aorta. Therefore, this study analyzed the prognostic values of IL-33, sST2, MPO, and MMP-9 plasma levels in patients with AAD at admission.

## 2. Materials and methods

### 2.1. Study design

A prospective cohort study was designed to explore the early prognostic values of IL-33, sST2, MPO, and MMP-9 in AAD. Research data was obtained from the Prospective Evaluation of Acute Chest Pain (PEACP) study. The PEACP study recruited patients with acute chest pain from tertiary hospitals in China and is registered at clinicaltrials.gov/ct2/home (Identifier: NCT04122573). Due to the PEACP study is primarily designed to establish the early risk stratification of acute myocardial infarction, and only one center collected blood samples and clinical data of AAD patients in a standardized way, thus this study was only included West China Hospital of Sichuan University. This study complied with the Declaration of Helsinki and all patients and/or their guardians signed informed consent forms. The ethics of this study were reviewed and approved by the Ethics Committee of the West China Hospital of Sichuan University (Identifier: 2019-565).

### 2.2. Study population

From December 2019 to January 2021, 5,843 patients visited the Chest Pain Center of the West China Hospital of Sichuan University, of which 259 adult patients were diagnosed with AAD according to their dissected aorta containing both true and false lumens. This was confirmed by CT or magnetic resonance angiography. Of these patients, 104 patients were excluded: 67 patients had an onset time greater than 3 days, 25 patients had other serious inflammatory diseases (such as solid tumors and blood system cancers and infections), and 12 patients failed to have a follow up. In total, 155 patients with AAD were included in the study.

### 2.3. Data collection

In this study, demographic characteristics, vital signs, medical history, treatment, laboratory examination, and imaging findings were recorded by two trained physicians using a structured electronic questionnaire. Laboratory examinations included complete blood cell count, blood biochemical examinations, cardiac markers, and coagulation index. Imaging findings included the aortic root diameter, maximum diameter of the thoracic aorta, maximum diameter of the abdominal aorta, minimum true lumen area, maximum false lumen area, thrombus status of the false lumen (no thrombus vs. partial thrombus vs. complete thrombus), and involved aortic branches (coronary artery, arch artery branch, arteria iliaca communis, renal artery, and mesenteric artery).

### 2.4. Immunoassays of plasma biomarkers

#### 2.4.1. ELISA assay of plasma IL-33, sST2, and MMP-9 concentrations

Venous blood was collected using EDTA, and plasma was collected after centrifugation and frozen at −80°C. The plasma concentrations of IL-33, sST2, and MMP-9 were determined using an enzyme-linked immunosorbent assay (ELISA). Following the manufacturer’s ELISA Kits’ instructions (Elabscience, E-EL-H2402c for IL-33, E-EL-H6082 for sST2, and E-EL-H6075 for MMP-9), 100 μl of standard, diluted standard, and sample, and sample dilution were added to the standard, blank, and sample wells, respectively. These were then incubated at room temperature for 90 min. The biotinylated antibody working solution (100 μl) was added to each well and incubated for 1 h. The plate was washed thrice, 100 μl of enzyme conjugate working solution was added, and the plate was incubated for 30 min. The plate was then washed five times, 90 μl substrate solution was added to each well, and incubated for a further 15 min. Fifty microliters of stop solution was added to each well, and the optical density of each well was measured at 450 nm using a microplate reader (REEDEA, Ledi Instrument Co., Ltd., China). A standard curve was drawn and the sample concentration was calculated.

#### 2.4.2. Chemiluminescence assay of plasma MPO concentrations

Myeloperoxidase was quantitatively detected using a magnetic particle chemiluminescence immunoassay. The MPO detection kit (HOB MY00041) and the reagent warehouse of an automatic chemiluminescence immunoanalyzer were used to automatically process all calibrators, quality control products, and test samples. Biotin-labeled antigens were mixed with specific immunoglobulin G (IgG) antibodies and magnetic particles coated with streptavidin (SA) in the samples to be tested. After washing, an enzyme-labeled anti-human IgG secondary antibody was added to form a solid-phase antigen-antibody enzyme-labeled secondary antibody complex, which was washed again to remove unbound enzyme-labeled antibodies and other substances. A luminescent substrate was added, which is catalyzed by an enzyme-labeled secondary antibody to emit photons. The number of photons emitted is directly proportional to the specific antibody content of the sample. The sample concentration was obtained by measuring these photons using an instrument.

### 2.5. Endpoint and follow-up

Approximately 6 months after a patient was discharged from the hospital, they were interviewed by trained physicians using structured questionnaires *via* telephone and return visits. In this study, the median follow-up period was 6.4 (5.2–7.4) months and the primary endpoint was all-cause mortality.

### 2.6. Statistical analysis

Sample size calculation: according to a preliminary experiment, the survival rate of patients in the low tertile ST2 group was approximately 90%, and the survival rate of patients in the high tertile ST2 group was approximately 50%. To meet α (type 1 error) < 0.01 and β (type 2 error) < 0.05, the calculated sample size was 110 cases. Assuming that the loss of follow-up rate was 10%, the minimum sample size required for this study was 122.

The patients were categorized into tertile (T1, T2, and T3) groups based on the biomarkers IL-33 (<12.8 vs. 12.8–16.8 vs. >16.8 pg/ml), sST2 (<43.0 vs. 43.0–88.3 vs. >88.3 ng/ml), MPO (<1.53 vs. 1.53–3.14 vs. >3.14 RU/ml), and MMP-9 (<8.8 vs. 8.8–16.7 vs. >16.7 ng/ml). Continuous variables with normal distributions are expressed as the mean ± standard deviation (SD) and were compared by Student’s *t*-test, while abnormally distributed continuous variables were reported as medians (25th–75th) and compared using the Mann–Whitney *U* test. Categorical variables were reported as frequencies (%) and compared using the χ^2^ test. Cox regression models were used to evaluate whether biomarkers were associated with time-to-mortality. The models were adjusted for age, gender (male vs. female), hypertension (yes vs. no), treatment (conservative treatment vs. surgery treatment), and classification (type A vs. type B). Multiplicative interactions analysis of these biomarkers in multivariate Cox regression models for all-cause mortality were conducted, and *P* for interaction <0.05 indicates a significant interaction between two biomarkers. The multivariate Cox regression models of all-cause mortality were conducted to analyze the prognostic evaluation value of these biomarkers in different subgroups, such as age (<55 vs. ≥55 years), time from admission to death (<5 vs. ≥5 days), treatment (conservative therapy vs. surgical therapy), and AAD classification (type A-AAD vs. type B-AAD), and *P* for interaction were tested. The cumulative survival rates of the three groups were calculated using the Kaplan–Meier curve and compared using the log-rank χ^2^ test. Pearson’s correlation analysis was used to detect correlations between biomarkers and imaging parameters. A two-sided *P*-value < 0.05 indicates statistical significance. Data were analyzed using SPSS Statistics version 26.0 (IBM Corp., Armonk, NY, USA) and R for Windows version 4.1.2 (Vienna, Austria).

## 3. Results

### 3.1. Baseline characteristics

In this study, the median age of the 155 patients with AAD was 55 (46–65) years; 30 (19.4%) were women; and 96 (61.9%) were classified as having type A-AAD. Of these patients, 41 died during the median follow-up of 6.4 (5.2–7.4) months. As shown in Table 1, the non-surviving patients were older; had fewer complications with hypertension; higher neutrophil counts, blood glucose levels, and D-dimer concentrations; lower platelet counts; and larger aortic root diameters and pseudolumen areas (all *P* < 0.05). Additionally, non-surviving patients were more likely to be classified as type A-AAD and more likely to receive conservative treatment compared to the surviving patients (*P* < 0.05).

**TABLE 1:** Baseline characteristics of patients with acute aortic dissection according to survival and death.

Variable	Survival *N* = 114 (73.5%)	Death *N* = 41 (26.5%)	*P*-value
Age, year	54.0 ± 12.0	58.7 ± 14.0	0.043
Female, *n* (%)	21 (18.4)	9 (22.0)	0.624
Current smoking, *n* (%)	57 (50.0)	19 (46.3)	0.688
Current drinking, *n* (%)	49 (43.0)	17 (41.5)	0.866
Systolic pressure, mmHg	146.8 ± 28.2	140.1 ± 29.6	0.194
Diastolic pressure, mmHg	83.5 ± 21.1	80.8 ± 20.9	0.477
Heart rate, beat/min	83.2 ± 16.6	81.2 ± 18.7	0.534
Oxygen saturation, %	97.8 ± 1.6	97.6 ± 1.9	0.619
**Chronic complications**			
Hypertension, *n* (%)	87 (76.3)	22 (53.7)	0.006
Diabetes mellitus, *n* (%)	5 (4.4)	4 (9.8)	0.207
Hyperlipidemia, *n* (%)	16 (14.0)	9 (22.0)	0.237
Coronary heart disease, *n* (%)	20 (17.5)	5 (12.2)	0.425
**Laboratory examination**			
Neutrophil count, 10^9^/L	10.2 ± 4.3	12.0 ± 3.4	0.018
Platelet count, 10^9^/L	165.6 ± 62.6	141.6 ± 55.5	0.034
Hemoglobin, g/L	133.3 ± 21.2	134.1 ± 14.4	0.842
Blood glucose, mmol/L	7.6 ± 2.3	8.9 ± 2.7	0.003
eGFR, ml/(min × 1.73 m^2^)	84.6 ± 24	79.7 ± 25.6	0.276
D-dimer, mg/L	5.65 (2.18–9.7)	18.36 (8.6–37.46)	<0.001
Hs-cTnT, ng/L	13.6 (8.8–23.4)	14.8 (11.0–29.6)	0.291
NT-proBNP, pg/ml	229 (110–920)	213 (98–695)	0.631
**Imaging examination**			
Number of branches involved	2 (1–4)	3 (2–5)	0.004
Diameter of aortic root, cm^2^	3.5 ± 0.4	3.7 ± 0.5	0.020
Maximum diameter of thoracic aorta, cm^2^	4.5 ± 1.0	5.0 ± 1.3	0.005
Maximum diameter of abdominal aorta, cm^2^	2.7 ± 0.5	2.7 ± 0.5	0.477
Maximum false cavity area, cm^2^	9.20 (6.82–12.75)	14.42 (10.88–16.99)	<0.001
Minimum true cavity area, cm^2^	1.21 (0.975–1.79)	1.35 (0.945–1.73)	0.792
Complete organization of thrombus, *n* (%)	14 (12.3)	5 (12.2)	0.989
Therapy, *n* (%)			<0.001
Conservative treatment	32 (28.1)	27 (65.9)	
Surgical treatment	82 (71.9)	14 (34.1)	
Classification, *n* (%)			0.001
Type A-AAD	62 (54.4)	34 (82.9)	
Type B-AAD	52 (45.6)	7 (17.1)	

The involved branches include coronary artery, brachiocephalic trunk, common carotid artery, subclavian artery, left and right renal artery, superior mesenteric artery, celiac trunk, common iliac artery, and external iliac artery.

### 3.2. Association between biomarkers and all-cause mortality

**FIGURE 1:**
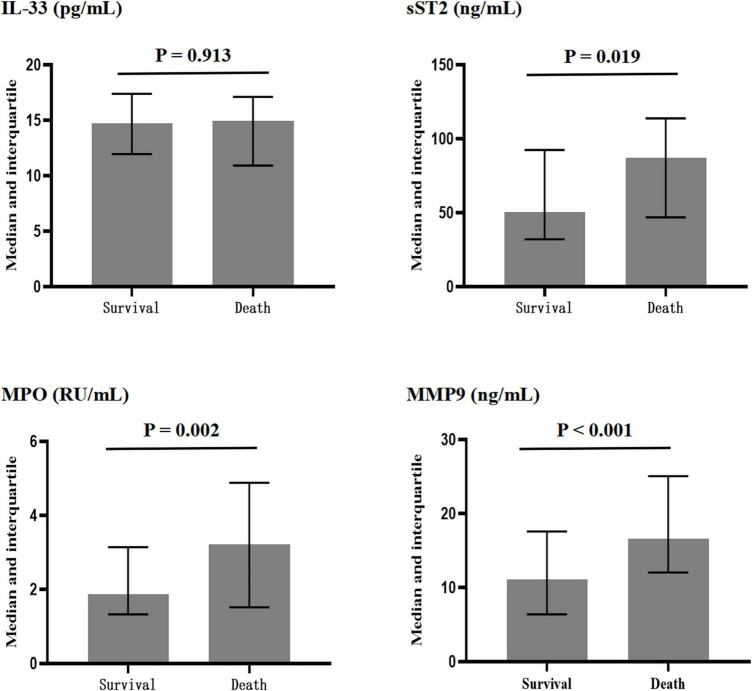
Plasma concentrations of interleukin-33 (IL-33), sST2, myeloperoxidase (MPO), and matrix metalloproteinase-9 (MMP-9) in surviving and dead patients.

As shown in Figure 1, compared with the surviving patients, the non-surviving patients had similar levels of IL-33 [15.0 (10.9–17.1) vs. 14.7 (12.0–17.4) pg/ml, *P* = 0.913]. However, the deceased patients had higher concentrations of sST2 [86.9 (46.9–113.8) vs. 50.3 (32.1–92.4) ng/ml, *P* = 0.019], MPO [3.22 (1.51–4.88) vs. 1.86 (1.32–3.14) RU/ml, *P* = 0.002], and MMP-9 [11.7 (6.4–17.6) vs. 16.6 (12.0–25.1) ng/ml, *P* < 0.001].

In the unadjusted Cox regression analysis (Table 2), the hazard ratio (HR) of the T3 tertile for all-cause mortality was 0.970 (95% CI: 0.462–2.035, *P* = 0.935), 3.945 (95% CI: 1.583–9.832, *P* = 0.003), 4.414 (95% CI: 1.886–10.328, *P* = 0.001), and 4.474 (95% CI: 1.668–11.996, *P* = 0.003) compared with T1 of IL-33, sST2, MPO, and MMP-9, respectively. After adjusting for demographic characteristics, vital signs, medical history, treatment, laboratory examination, and imaging findings, the HR (T3 vs. T1) was 0.870 (95% CI: 0.412–1.836, *P* = 0.714), 3.769 (95% CI: 1.504–9.446, *P* = 0.005), 4.689 (95% CI: 1.985–11.076, *P* < 0.001), and 4.748 (95% CI: 1.763–12.784, *P* = 0.002) for IL-33, sST2, MPO, and MMP-9, respectively. In addition, sST2, MPO, and MMP-9 levels increased by 1 SD, and the HR for mortality increased to 1.415 (95% CI: 1.094–1.896, *P* = 0.009), 1.554 (95% CI: 1.250–2.133, *P* < 0.001), and 1.563 (95% CI: 1.220–2.043, *P* = 0.001), respectively.

**TABLE 2:** Cox regression analysis of the relationship between biomarkers and all-cause mortality in patients with acute aortic dissection.

Markers	Unadjusted		Adjusted	
	HR (95% CI)	*P*	HR (95% CI)	*P*
IL-33		0.995		0.906
T1	Reference	–	Reference	
T2	1.005 (0.469–2.150)	0.991	1.019 (0.476–2.181)	0.962
T3	0.970 (0.462–2.035)	0.935	0.870 (0.412–1.836)	0.714
Per SD	0.940 (0.691–1.277)	0.691	0.913 (0.601–1.340)	0.623
sST2		0.013		0.017
T1	Reference	–	Reference	
T2	2.867 (1.112–7.397)	0.029	3.083 (1.182–8.043)	0.021
T3	3.945 (1.583–9.832)	0.003	3.769 (1.504–9.446)	0.005
Per SD	1.322 (1.044–1.673)	0.020	1.415 (1.094–1.896)	0.009
MPO		0.001		<0.001
T1	Reference	–	Reference	
T2	1.638 (0.635–4.230)	0.308	1.734 (0.670–4.487)	0.257
T3	4.414 (1.886–10.328)	0.001	4.689 (1.985–11.076)	<0.001
Per SD	1.701 (1.330–2.175)	<0.001	1.554 (1.250–2.133)	<0.001
MMP-9		0.011		0.008
T1	Reference	–	Reference	
T2	3.617 (1.334–9.805)	0.012	3.797 (1.398–10.315)	0.009
T3	4.474 (1.668–11.996)	0.003	4.748 (1.763–12.784)	0.002
Per SD	1.606 (1.247–2.070)	<0.001	1.563 (1.220–2.043)	0.001

The models were adjusted by age, gender (male vs. female), hypertension (yes vs. no), treatment (conservative treatment vs. surgery treatment), classification (type A vs. type B). Other unexplained covariables are continuous variables. *P* for interactions were tested in multivariable Cox-regression models. IL-33, interleukin-33; MPO, myeloperoxidase; MMP-9, matrix metalloproteinase-9; HR, hazard ratio; CI, confidence interval; SD, standard deviation.

**FIGURE 2:**
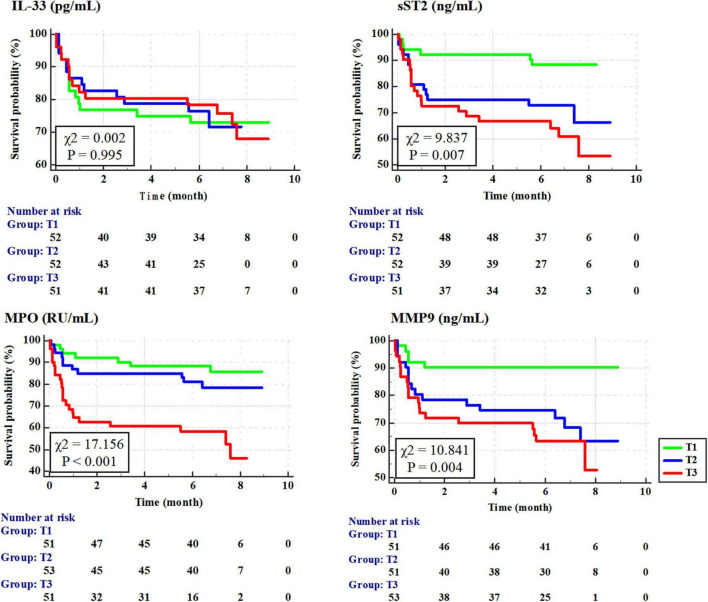
Cumulative survival rate of patients with tertile of interleukin-33 (IL-33), sST2, myeloperoxidase (MPO), and matrix metalloproteinase-9 (MMP-9) according to Kaplan–Meier survival curves.

According to the Kaplan–Meier survival analysis (Figure 2), the cumulative survival rates of T1 vs. T2 vs. T3 for sST2, MPO, and MMP-9 were 88.3 vs. 66.9 vs. 54.2% (*P* = 0.007), 85.1 vs. 79.0 vs. 46.4% (*P* < 0.001), and 90.0 vs. 63.5 vs. 53.1% (*P* = 0.004), respectively.

### 3.3. Subgroup analysis

**FIGURE 3:**
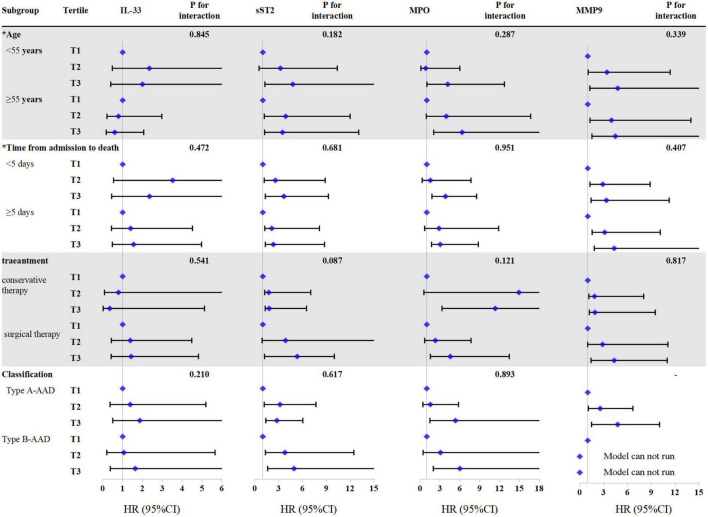
Cox regression analysis of all-cause mortality in different subgroups according to interleukin-33 (IL-33), sST2, myeloperoxidase (MPO), and matrix metalloproteinase-9 (MMP-9). *Variables are classified according to the median. There is no mortality case in type B-AAD patients with lowest tertile of MMP-9, the COX regression model of this group cannot be run. The models were adjusted by age, gender, hypertension, treatment, and classification.

According to the results (Figure 3), association of sST2, MPO, and MMP-9 with all-cause mortality were consistent in the subgroup of age (<55 vs. ≥55 years), time from admission to death (<5 vs. ≥5 days), treatment (conservative therapy vs. surgical therapy), and AAD classification (type A-AAD vs. type B-AAD) (*P* for interaction ≥0.05).

### 3.4. Correlation and interactions analysis

**FIGURE 4:**
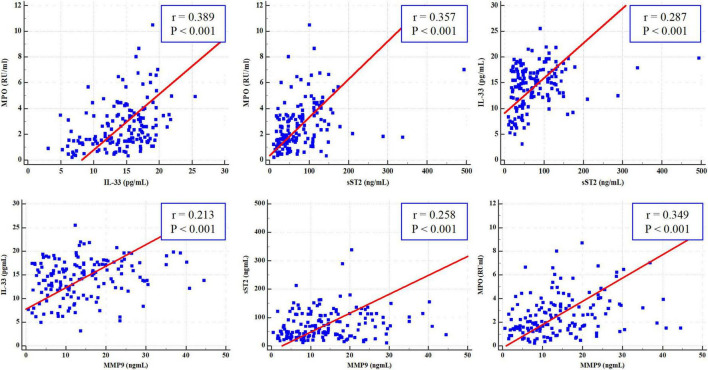
Pearson correlation analysis of interleukin-33 (IL-33), sST2, myeloperoxidase (MPO), and matrix metalloproteinase-9 (MMP-9).

According to the Pearson correlation analysis (Figure 4), there was a significant positive correlation between IL-33 and MPO (*r* = 0.389, *P* < 0.001), IL-33 and sST2 (*r* = 0.287, *P* < 0.001), sST2 and MPO (*r* = 0.357, *P* < 0.001), IL-33 and MMP-9 (*r* = 0.213, *P* < 0.001), sST2 and MMP-9 (*r* = 0.258, *P* < 0.001), and MPO and MMP-9 (*r* = 0.349, *P* < 0.001).

Considering that these biomarkers are significantly intercorrelated, interactions analysis in multivariate Cox regression models for all-cause mortality were conducted. As shown in Table 3, there was a significant multiplicative interaction between sST2 and MPO (HR: 1.732, 95% CI: 1.231–2.496, *P* = 0.001) for all-cause mortality. While, no other significant multiplicative interactions were observed (all *P* ≥ 0.05; Table 3).

**TABLE 3:** Multiplicative interactions analysis of biomarkers in multivariate Cox regression models for all-cause mortality.

Markers	sST2		MPO		MMP-9	
	HR (95% CI)	*P* for interaction	HR (95% CI)	*P* for interaction	HR (95% CI)	*P* for interaction
IL-33	1.207 (0.923–1.578)	0.170	1.307 (0.980–1.741)	0.068	0.930 (0.684–1.265)	0.644
sST2	–	–	1.732 (1.231–2.496)	0.001	0.961 (0.780–1.185)	0.711
MPO	–	–	–	–	1.007 (0.823–1.232)	0.944

The models were adjusted by age, gender (male vs. female), hypertension (yes vs. no), treatment (conservative treatment vs. surgery treatment), classification (type A vs. type B). IL-33, interleukin-33; MPO, myeloperoxidase; MMP-9, matrix metalloproteinase-9; HR, hazard ratio; CI, confidence interval.

Moreover, Pearson correlation analysis showed that the biomarkers sST2, MPO, and MMP-9 had a significant positive correlation with aortic root diameter, maximum diameter of the thoracic aorta, and maximum pseudolumen area (all *P* < 0.001; Table 4).

**TABLE 4:** Pearson correlation analysis of IL-33, sST2, MPO, and MMP-9 with aortic anatomical parameters.

Variable	IL-33 (pg/ml)	sST2 (ng/ml)	MPO (RU/ml)	MMP-9 (ng/ml)
Diameter of aortic root	*r* = 0.007	*r* = 0.312***	*r* = 0.284***	*r* = 0.440***
Maximum diameter of thoracic aorta	*r* = 0.021	*r* = 0.229***	*r* = 0.205***	*r* = 0.383***
Maximum diameter of abdominal aorta	*r* = 0.039	*r* = 0.101*	*r* = 0.095*	*r* = 0.203*
Minimum true cavity area	*r* = 0.048	*r* = 0.059	*r* = 0.025	*r* = 0.106*
Maximum false cavity area	*r* = 0.093*	*r* = 0.417***	*r* = 0.483***	*r* = 0.369***
Number of branches involved	*r* = 0.033	*r* = 0.169**	*r* = 0.241***	*r* = 0.127**

The involved branches include coronary artery, brachiocephalic trunk, common carotid artery, subclavian artery, left and right renal artery, superior mesenteric artery, celiac trunk, common iliac artery, and external iliac artery. IL-33, interleukin-33; MPO, myeloperoxidase; MMP-9, matrix metalloproteinase-9.

*P < 0.05; **P < 0.01; ***P < 0.001.

## 4. Discussion

This study investigated the association between neutrophil-related inflammatory markers (IL-33, sST2, MPO, and MMP-9) and all-cause mortality in patients with AAD. Our findings demonstrated that elevated plasma concentrations of sST2, MPO, and MMP-9 were independently associated with 6-month mortality in patients with AAD. Further subgroup analysis confirmed the application value of these biomarkers in early assessment in different ages, treatment, and classification. While, IL-33 levels at admission were consistent between surviving and non-surviving patients. This study emphasized that the biomarkers sST2, MPO, and MMP-9 can not only be used to evaluate the prognosis of patients with AAD, but also reflect the disease severity; thus, these biomarkers are useful indicators for clinical practice at chest pain centers.

Myeloperoxidase catalyzes the formation of oxidants at specific positions in leukocytes and participates in inflammation and oxidative stress. Brennan et al. evaluated the clinical value of MPO in predicting adverse events in patients with acute chest pain (21). As reported, patients with high MPO levels (highest quartile vs. lowest quartile) were more likely to have major adverse cardiac events at 30 days (odds ratio: 4.7, 95% CI: 2.7–8.8) and 6 months (odds ratio: 4.7, 95% CI: 2.9–7.7). Similarly, our results showed that AAD patients in the highest tertile of MPO had a fourfold higher risk of mortality than those in the lowest tertile of MPO.

Matrix metalloproteinase is a zinc enzyme responsible for degrading extracellular matrix components, including fibronectin, collagen, elastin, and proteoglycans. High expression of soluble MMP-9 can be measured in the plasma of patients with abdominal aortic aneurysm or aortic dissection (22–25). Similar to MPO, MMP-9, as a neutrophil-derived mediator, can effectively predict the mortality risk of patients with AAD.

Previous studies have repeatedly confirmed that sST2 can not only effectively diagnose heart failure but also provide prognostic information independent of traditional evaluation indicators (26, 27). Recent study have emphasized the high predictive value of sST2 in the early diagnosis of AAD (area under the receiver operating characteristic curve: 0.97) (14). Our study further proves that sST2 is an independent factor for mortality in patients with AADs. However, although IL-33 is a ligand of sST2, IL-33 cannot effectively predict the progression of AAD. The differences in prognostic evaluation are currently difficult to explain, and further studies are needed. Although many studies have discussed the relationship between sST2 and inflammatory diseases, only a few have analyzed the relationship between IL-33 and sST2 with disease severity and outcome. In a study on coronary heart disease, serum levels of IL-33 were not associated with mortality, although ST-segment elevation myocardial infarction patients with high IL-33 levels had a higher risk of mortality (28). However, contrary to sST2, a low level of IL-33, is associated with a higher mortality risk in critically ill patients (29). Similar to our results, patients with COPD had comparable IL-33 levels regardless of disease severity, but pulmonary function was inversely correlated with sST2 (30). These results were consistent with severe alcoholic hepatitis (31).

Aortic lesions directly reflect the severity of AAD and are closely related to its prognosis (32, 33). Our results showed that sST2, MPO, and MMP-9 levels were significantly positively correlated with aortic diameter, pseudolumen area, and involved branches. These results emphasize that sST2, MPO, and MMP-9 levels reflect the degree of vascular lesions and tears and thus, these biomarkers are useful indicators for early risk stratification in patients with AAD. An interesting finding is that there was a significant multiplicative interaction between sST2 and MPO. This results indicates that patients with elevated both sST2 and MPO have a higher risk of mortality, thus sST2 and MPO may play a synergistic role in development of AAD.

Considering that an increasing number of studies have discussed the proinflammatory effect of IL-33/sST2 on neutrophils, our study systematically detected the related markers, IL-33, sST2, and neutrophil granzyme (MPO and MMP-9). Similar to a previous study, IL-33 was significantly positively correlated with sST2 levels in patients with diabetes (*r* = 0.44). Previous studies have also found that elevated sST2 levels in circulation are related to changes in IL-33 levels in tissues (34). This explains why IL-33 and sST2 levels are positively correlated, although they have opposing physiological functions. Considering that IL-33 levels are commonly higher in the diseased population than in the healthy population, high expression of sST2 may limit the function of IL-33 by exerting an antagonistic effect. Therefore, sST2 levels are positively associated with cardiovascular disease severity, but this does not mean that IL-33 is always beneficial (34). Moreover, there were significant correlations between IL-33/sST2 and neutrophil granzyme (MPO and MMP-9) levels, suggesting that IL-33 promotes the release of granzyme from neutrophils, which may be involved in the pathophysiology of AAD. Further cellular and animal experiments are required to confirm this hypothesis.

This study had some limitations. As this was a single-center study with a relatively small sample size, the universality of our findings requires more large-scale prospective multicenter studies to verify. Additionally, neutrophil enzyme activity was not detected in this study, and measurement of enzyme activity may provide a stronger predictive value. Moreover, the dynamic detection and analysis of markers during disease progression may provide more prognostic information. Finally, this study did not collect data on other adverse events during hospitalization and failed to identify the specific cause of death in patients.

## 5. Conclusion

The blood biomarkers sST2, MPO, and MMP-9 were independently associated with 6-month mortality in patients with AAD. The significant associations of sST2, MPO, and MMP-9 with aortic anatomical parameters indicate that these biomarkers are useful for early risk stratification in patients with AAD. However, patients with AAD had comparable IL-33 levels regardless of disease severity. A significant positive correlation between these biomarkers suggests a pathogenic role for the IL-33/sST2/neutrophil granzyme system in patients with AAD.

## Data availability statement

The raw data supporting the conclusions of this article will be made available by the authors, without undue reservation.

## Ethics statement

The studies involving human participants were reviewed and approved by the Ethics Committee of the West China Hospital of Sichuan University. The patients/participants provided their written informed consent to participate in this study.

## Author contributions

YJ, DL, and RZ designed the research. DL, YJ, and YL analyzed the data under the supervision of XL. DL and YJ wrote the first draft of the manuscript. JY, YL, FL, WL, WJ, XL, and ZW reviewed the manuscript and provided critical scientific input. ZW had main responsibility for the final content of the manuscript. All authors approved the final draft of the manuscript.
